# Evolution of Pallial Areas and Networks Involved in Sociality: Comparison Between Mammals and Sauropsids

**DOI:** 10.3389/fphys.2019.00894

**Published:** 2019-07-12

**Authors:** Loreta Medina, Antonio Abellán, Ester Desfilis

**Affiliations:** Department of Experimental Medicine, Institut de Recerca Biomèdica de Lleida - Fundació Dr. Pifarré (IRBLleida), University of Lleida, Lleida, Spain

**Keywords:** medial amygdala, BST, social cognition, affiliation, dorsal ventricular ridge, six part pallial model, orbito frontal cortex, pallial amygdala

## Abstract

Birds are extremely interesting animals for studying the neurobiological basis of cognition and its evolution. They include species that are highly social and show high cognitive capabilities. Moreover, birds rely more on visual and auditory cues than on olfaction for social behavior and cognition, just like primates. In primates, there are two major brain networks associated to sociality: (1) one related to perception and decision-making, involving the pallial amygdala (with the basolateral complex as a major component), the temporal and temporoparietal neocortex, and the orbitofrontal cortex; (2) another one related to affiliation, including the medial extended amygdala, the ventromedial prefrontal and anterior cingulate cortices, the ventromedial striatum (largely nucleus accumbens), and the ventromedial hypothalamus. In this account, we used an evolutionary developmental neurobiology approach, in combination with published comparative connectivity and functional data, to identify areas and functional networks in the sauropsidian brain comparable to those of mammals that are related to decision-making and affiliation. Both in mammals and sauropsids, there is an important interaction between these networks by way of cross projections between areas of both systems.

## Introduction

In primates, several studies have shown a correlation between social network size and the volumes of specific parts of the telencephalic pallium, including the orbitofrontal cortex, the cortical and basolateral amygdala, the temporo-parietal cortical junction, and the superior temporal sulcus (Lewis and Barton, 2006; Powell et al., 2010, 2012; Bickart et al., 2011; Kanai et al., 2011). In contrast, such a correlation is not observed when other neocortical areas and the hippocampal formation are considered. One important aspect to remark is that areas related to sociality are reciprocally connected and are clustered together in functional MRI (fMRI) studies (Bickart et al., 2012). In particular, fMRI in humans described two distinct brain networks positively correlated to sociality, one involved in social perception (the impressions and inferences that one individual of a group makes on another, from selective attention to inferences of intentionality) and another one involved in social affiliation (the association of individuals within a group, including social attachment) (Byrne and Bates, 2007; Bickart et al., 2012). The network related to social perception involves the “ventrolateral amygdala” (including the pallial amygdala, encompassing the cortical and basolateral or basal amygdala complex), the fusiform gyrus (for face recognition) and other temporal neocortical areas, and the orbitofrontal cortex; the network related to social affiliation involves the medial amygdala, the ventromedial prefrontal cortex and adjacent subgenual and anterior cingulate cortical areas, the ventromedial striatum (largely including nucleus accumbens), and the ventromedial hypothalamus (Bickart et al., 2012).

While social cognition in primates relies more on visual and auditory cues, and less so on olfactory cues, the opposite is true in most non-primate mammals. Comparative studies of brain areas and networks involved in sociality are important because: (1) they can identify useful non-primate models for studying in depth the neural basis of social cognition at molecular, cellular, and circuit levels, (2) they can help to identify selective pressures and developmental mechanisms behind evolutionary change (MacLean, 2016), and (3) they can help to identify minimal neural requirements for achieving sophisticated cognitive tasks, such as predict intentionality of others and other aspects of theory of mind. In this sense, birds are extremely interesting animals because some species are highly social, show high cognitive capabilities, use vocalization in social contexts (a capacity that is particularly well developed in songbirds; Hessler and Doupe, 1999), and include at least a family (corvids) with capacity for physical reasoning, for remembering the past and plan for the future and for thinking about another’s perspective (Clayton and Emery, 2015; Boucherie et al., 2019). In addition, birds – like primates – rely more on visual and auditory cues than on olfaction for social behavior and cognition (Clayton and Emery, 2015; Mayer et al., 2017). Thus, although sophisticated aspects of social cognition in corvids and primates were achieved by independent evolution, birds are good models for studies on the neural basis of sociality and its evolution. However, the pallium of birds and mammals have greatly diverged during evolution, which has made the comparison of pallial areas and interpretations quite controversial (discussed by Reiner et al., 2004). For inter-species brain comparisons to make sense, it is mandatory to first unravel the brain building plan (Bauplan or morphoplan), which is shared by different vertebrates. Developmental studies, particularly those on combinatorial expression patterns of early regulatory genes in relation to the topological framework of the neural tube, have become extremely useful to unravel the brain morphoplan, with its basic divisions comparable across vertebrates (Nieuwenhuys and Puelles, 2016). The conclusions of this type of approach support that a large lateroventral part of the avian and reptilian pallium (called the dorsal ventricular ridge) derives from pallial embryonic divisions that gives rise to the pallial amygdala and other areas of the so-called piriform lobe in mammals (Puelles et al., 2000, 2017; Medina et al., 2011, 2017a; Abellán et al., 2013; Desfilis et al., 2018), a proposal also supported by results of fate mapping (Hirata et al., 2009; Soma et al., 2009; Waclaw et al., 2010; Bupesh et al., 2011; Puelles et al., 2016a; García-Moreno et al., 2018; Rueda-Alaña et al., 2018), tract-tracing studies (Bruce and Neary, 1995; Martínez-García et al., 2007), and more recently, by single-cell transcriptome (Tosches et al., 2018). In this account, we will review the different data pointing to the presence of a pallial amygdala-like region in the sauropsidian dorsal ventricular ridge, as well as the possible existence of an area comparable to the orbitofrontal cortex. We will also review the known connections and functions of these areas and discuss their putative implication in social cognition.

## The Evolutionary Developmental Biology Approach to Unravel the Pallium Morphoplan

Understanding the evolutionary origin of the neocortex has inspired a vast amount of research and still generates vehement and thrilling debates as well as new publications, often with different conclusions (for example: Karten, 1997; Puelles, 2001; Butler et al., 2011; Dugas-Ford et al., 2012; Belgard et al., 2013; Tosches et al., 2018). The neocortex derives from the telencephalic pallium, but the continuing controversy on its evolution relates to the uncertainty on how many pallial divisions there are and how they are compared across vertebrates. The traditional comparative neuroanatomy approach, excellent for analyzing the gradual evolutionary variations in relatively well conserved brain areas and fiber tracts (for example, the basal ganglia and the dopaminergic nigrostriatal projection; Reiner et al., 1998), have not provided a satisfactory answer to the question on the evolutionary origin of the neocortex, which has involved a high degree of divergence. The evolutionary developmental biology (evodevo) approach, integrated as part of the extended evolutionary synthesis, is complementary to the traditional approaches for understanding evolution and has the power of providing developmentally based explanations of the organism (or organ) form and function, as well as new venues for both exploring the mechanisms behind innovation and answering the question of how novel complex traits originate (Hall, 2003; Moczek et al., 2015). The latter often relates to developmental changes in genetic networks involved in patterning, specification, cell proliferation, and/or other aspects of development, which emerge and spread in populations under favorable conditions for ecological (natural *sensu* Darwin) or sexual selection (i.e., if the variation provides advantage for survival and/or reproduction).

The evodevo approach is successfully being applied to study brain evolution, providing highly powerful tools for: (1) understanding the brain morphoplan and comparing its basic building blocks or divisions across species (Puelles et al., 2000; Medina and Abellán, 2009; Moreno et al., 2010; González et al., 2014), (2) identifying hidden or obscure cases of deep homology, or partial homology (Shubin et al., 2009), (3) identifying homologous brain areas and networks involved in particular functions and behaviors, such as those related to sociality (Medina et al., 2011; O’Connell, 2013), (4) unraveling the genetic and developmental mechanisms involved in convergence (Pfenning et al., 2014; Whitney et al., 2014) and in evolutionary variations (for example, eye loss in blind cavefish: McGaugh et al., 2014; variations in optic tectum size: Striedter and Charvet, 2008). Importantly, homologous brain divisions typically show similar combinatorial expression patterns of highly conserved transcription factors and other regulatory proteins during early (phylotypic) stages of development of different vertebrates (Puelles et al., 2000; Puelles and Medina, 2002; Medina, 2007; Nieuwenhuys and Puelles, 2016). Variations in expression patterns of developmental regulatory genes occur more often during late development and are behind cases of morphological divergence (Medina et al., 2013).

Puelles and colleagues were the first in using this approach to identify the same basic building divisions in the embryonic telencephalon of mouse and chicken (Puelles et al., 2000). In the pallium, they identified a novel ventral pallial division (VP) as a partition of the classical piriform lobe or lateral pallium, leading to the proposal of a tetrapartite model of pallial divisions, with medial, dorsal, lateral and ventral pallia (this proposal was recently revisited by Puelles et al., 2017). According to the initial proposal, the VP of mouse and chicken showed expression of pan-pallial transcription factor-related genes (such as Pax6 in the ventricular zone and Tbr1 in the mantle), but lacked ventricular zone expression of Emx1 (typical in the rest of the pallium) and Dlx2 (typical of the subpallium) (Puelles et al., 2000). This division was located just above the pallio-subpallial boundary and showed the olfactory tract at its surface (Puelles et al., 2000; Puelles, 2001). This initial proposal was reinforced later by the identification in mouse of transcription factors expressed in the ventral pallium but not in adjacent pallial and subpallial divisions (such as Dbx1, expressed in the VP ventricular zone, but not in the dorsal pallium nor the subpallial ganglionic eminences; and Lhx9, highly expressed in the VP mantle, but not in the subpallial striatum/pallidum and only transitorily in the dorsal pallium outer layer, in relation to Cajal-Retzius cells) (Dbx1: Yun et al., 2001; Medina et al., 2004; Lhx9: Rétaux et al., 1999; Abellán et al., 2009; Medina et al., 2011). The VP has also been identified in non-mammals on the basis of Lhx9 expression, in combination with other regulatory genes, although this division does not express Dbx1 in non-mammals (Moreno et al., 2004; Abellán et al., 2009; Medina et al., 2011; Vicario et al., 2017; Desfilis et al., 2018).

According to the tetrapartite pallial model, the VP includes the olfactory bulb at its rostral pole, followed caudally by olfactory pallial areas in all vertebrates (Puelles, 2001; revisited by Puelles et al., 2017). In mammals, this includes the olfactory cortex, and more caudally the cortical amygdalar areas, all of which receive input from the main and/or accessory olfactory bulbs (Puelles, 2001; Puelles et al., 2017). It also includes several nuclei located deep (in topological terms; i.e., considering the radial dimension) to the olfacto-recipient cortical areas, such as the endopiriform nuclei and the basal amygdalar complex (Puelles et al., 2017). In birds and reptiles, it produces a ventral part of the dorsal ventricular ridge (DVR), which in birds includes the so-called nidopallium and the arcopallium, both of which are located deep to some olfactory superficial areas (Puelles et al., 2017).

However, although the existence of a VP sector in the pallium is currently unquestionable, its rostrocaudal extension and number of derivatives are currently a matter of debate. First, the VP derivatives have been clearly traced in mice using migration assays (Soma et al., 2009; Bupesh et al., 2011) and Dbx1 cell lineage tracing in transgenic reporter mice (Hirata et al., 2009; Waclaw et al., 2010; Puelles et al., 2016a). According to these results, the VP progenitors give rise to the ventral parts of the piriform cortex and endopiriform nuclei (but not their dorsal parts) and to large part of the pallial amygdala except its caudal pole (Puelles et al., 2016a). Second, in birds and reptiles, VP derivatives would not include the caudal DVR, encompassing the avian arcopallium and the reptilian dorsolateral amygdala and nucleus sphericus, which show discrepant expression patterns with those of the nidopallium/anterior DVR during development (Abellán et al., 2014; Medina et al., 2017a; Desfilis et al., 2018). Third, based on cell lineage tracing and gene expression patterns, the VP derivatives do not include the whole olfactory bulbs, but only part of them (as discussed by Desfilis et al., 2018).

## Toward a Model of Six Pallial Divisions?

The controversial data exposed above on the VP extension and derivatives, together with the difficulty found when trying to compare the embryonic pallium of mammals, birds and reptiles using gene expression patterns, prompted us to propose an alternative model of six pallial divisions, which is currently under evaluation (Medina et al., 2017a; Desfilis et al., 2018). The six part pallial model takes into consideration the experimental and genetic cell lineage results on VP derivatives in mouse, together with the gene expression patterns that are discrepant with a VP profile in different vertebrates (for example, expression of Emx1 in the ventricular zone, which happens in the rostral and caudal poles) in order to better delimit the VP and its derivatives (Medina et al., 2017a; Desfilis et al., 2018). According to the six part pallial model, the lateral pallium (LP) includes the dorsal part of the piriform lobe (the latter is neatly delineated by Lmo3 expression, encompassing VP and LP; Abellán et al., 2009). In contrast to VP, LP is poor in Dbx1-lineage cells (Puelles et al., 2016a) and Lhx9 (Abellán et al., 2009) and includes the dorsal parts of the olfactory cortex and endopiriform nuclei (Abellán et al., 2009, 2014; Desfilis et al., 2018). In birds, the LP includes the so-called mesopallium, which expresses Lmo3 but not Lhx9 in the mantle (Abellán et al., 2009).

**Figure 1:**
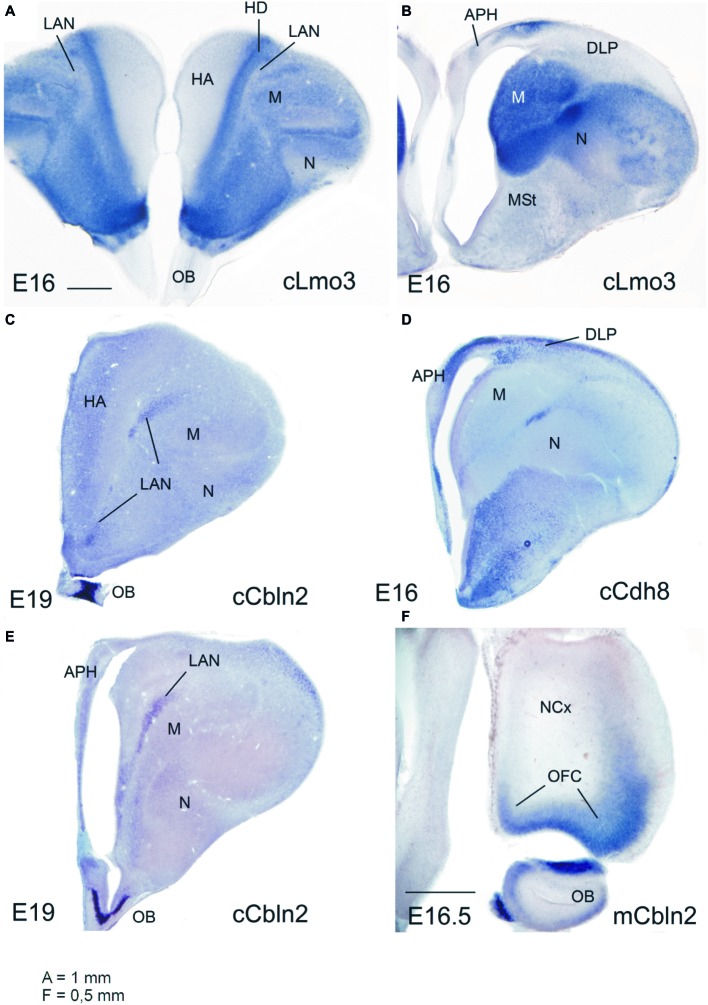
Expression of the mRNA of selected regulatory genes in the embryonic telencephalon of chicken and mouse. **(A,B)** Lmo3 expression in chicken (E16): like in mammals, this is rich in the ventral pallium (nidopallium, N) and lateral pallium (mesopallium, M), in parts of the dorsal pallium (densocellular hyperpallium, HD) and parts of the medial pallium (parahippocampal area, APH). In contrast, it is extremely weak in the dorsolateral pallium (DLP), including its rostral extension (laminar pallial nucleus or LAN). **(C,E)** Cbln2 expression in the chicken (E19): note the distinct expression in the LAN, resembling that in the mouse orbitofrontal cortex (OFC). **(D)** Cdh8 expression in chicken (E16): note the expression in the DLP, resembling that in the mouse claustrum (which is also part of DLP). **(F)** Cbln2 expression in the OFC of E16.5 mouse (which belongs to the rostral pole of the mouse DLP). Other abbreviations: HA, apical hyperpallium; MSt, medial striatum; OB, olfactory bulb. Scale bar: A = 1 mm (applies to **A–E**). *F* = 0.5 mm.

Dorsal to VP and LP, the six part pallial model proposes the existence of a different pallial sector, which is also distinct from the dorsal pallium and is called the dorsolateral pallium (DLP; Abellán et al., 2014; Medina et al., 2017a). This division is distinguished from early developmental stages in different amniotes, as a sector showing moderate to high expression of Emx1, Lhx2, and Lhx9 in the ventricular zone and/or mantle (Abellán et al., 2009, 2014; Desfilis et al., 2018), and it extends from rostral to caudal levels (Desfilis et al., 2018). In mouse, the DLP also shows strong expression of Nr4a2/Nurr1 from early stages and includes the claustroinsular complex (Watson and Puelles, 2017; discussed by Desfilis et al., 2018). Note that this complex is attributed to the LP and compared to the avian mesopallium in the current version of the tetrapartite pallial model (Puelles et al., 2016b, 2017; Watson and Puelles, 2017; see also Smith et al., 2018, where this sector is compared between different mammals), but the problem with this proposal is that while the mesopallium is part of the piriform lobe and rich in Lmo3, the claustroinsular complex is above and poor in Lmo3 (see data in Abellán et al., 2009) (Figure 1B). Thus, according to the six part pallial model, the claustroinsular complex is not comparable to the mesopallium and is not part of the lateral pallium, but belongs to the DLP. The DLP also extends rostrally into the olfactory bulb (Desfilis et al., 2018) and caudally contains the lateral entorhinal cortex in mammals and comparable areas in sauropsids (Abellán et al., 2014; Medina et al., 2017b; Desfilis et al., 2018).

Caudal to VP, the six part pallial model proposes the existence of another pallial division, the ventrocaudal pallium (VCP), which is rich in expression of Lhx2, Lhx9, and Emx1 in the ventricular zone and mantle (Medina et al., 2017a; Desfilis et al., 2018). In chicken and lizard, this division is clearly distinguished from early stages and relates to a ventricular sector located caudally, which is easier to visualize in sagittal or horizontal sections (Abellán et al., 2009; Desfilis et al., 2018). In chicken, the VCP includes the arcopallium; while in the long-tailed lacertid lizard, it includes the dorsolateral amygdala and nucleus sphericus (Medina et al., 2017a; Desfilis et al., 2018). In chicken and lizards, the VCP is clearly separated from VP by a cell poor lamina, giving additional support for the distinction of VCP as a major division and not simply a subdivision of VP (discussed by Desfilis et al., 2018). Note that cell poor glial palisades as the one that separates VP and VCP are only observed in the boundary between major divisions, such as pallium versus subpallium, nidopallium versus mesopallium, mesopallium from DLP, and DLP from hyperpallium, but not between internal subdivisions within major regions as the nidopallium or mesopallium. In mouse, the VCP is also distinguished in the caudalmost pallium from early stages, characterized for its strong expression of Lhx2, Lhx9, and Emx1, and because it lacks ventricular zone expression of Dbx1 (Medina et al., 2017a). The VCP of mouse appears to give rise to the caudal pole of the pallial amygdala (Medina et al., 2017a).

As noted above, more studies are needed to evaluate the validity of this six part pallial model or morphoplan in amniotes and, if so, to investigate which of the six divisions are present in anamniotes. However, we believe that the six pallial divisions proposed in the model are truly independent entities based on their distinct embryonic genoarchitecture from early stages, unique topological position, separation by cell-poor laminae, and presence across different amniotes, including mammals, birds, and reptiles (Desfilis et al., 2018). Moreover, so far, we found that the use of this morphoplan can be highly useful for comparative purposes and will help to decipher the evolutionary origin of the pallial areas or regions found in different vertebrates. In the context of this article, it can help to investigate the presence in non-mammals of areas homologous to the pallial amygdala and the orbitofrontal cortex, which in mammals are an important part of the brain networks involved in social cognition.

## The Pallial Amygdala Across Vertebrates

According to the six part pallial model, in mammals, the pallial amygdala derives from the VP and the VCP (Medina et al., 2017a). The VP gives rise to the nidopallium in birds and a large part of the DVR in reptiles, while the VCP produces the arcopallium in birds and the dorsolateral amygdala and nucleus sphericus in reptiles (Desfilis et al., 2018). The avian nidopallium and the reptilian DVR have often been compared to the neocortex or to specific cell subpopulations of the neocortex (Reiner, 1991; Butler, 1994a; Karten, 1997; Dugas-Ford et al., 2012; Briscoe and Ragsdale, 2018). These various proposals consider that, even though the sauropsidian DVR and the mammalian neocortex possess many non-homologous, divergent areas, they also contain a conserved set of neuron subtypes and connections, which are considered homologous (Karten, 1997; Dugas-Ford et al., 2012; Briscoe and Ragsdale, 2018). The problem with this is that the DVR and the neocortex originate in different embryonic compartments of the pallium, as noted above (see also discussions on this subject by Puelles, 2001; Aboitiz et al., 2003; Medina et al., 2013). However, a major problem is that the pallia of sauropsids and mammals have undergone greatly divergent routes, and the derivatives of the same (homologous) embryonic compartments of the pallium are highly dissimilar in sauropsids and mammals. This is in line with analysis of the transcriptome (including expression of more than 5,000 genes) of the different pallial regions, which do not support homology of adult derivatives of pallial compartments between sauropsids and mammals (with the only exception of the hippocampal formation, derived from the medial pallium) (Belgard et al., 2013). According to these data, regions that share the same embryonic origin exhibit no greater transcriptomic similarity than regions derived from different embryonic sectors, such as the chicken nidopallium and mouse neocortex (Belgard et al., 2013). However, when single-cell transcriptome (by mRNA sequencing) has been carried out, which allows selection of only glutamatergic neurons of pallial lineage (discarding interneurons of subpallial origin and glial cells), the results have been different and show unequivocal similarity between the turtle posterior DVR and the mouse pallial amygdala, with the only exception of the lateral nucleus of the amygdala, which correlates with the anterior DVR (Tosches et al., 2018). By contrast, these data did not support the cell-type homology between turtle DVR and mammalian neocortex (Tosches et al., 2018), although parts of neocortex and DVR show some spectacular cases of evolutionary convergence (such as that between the avian caudal nidopallium and the mammalian prefrontal cortex; Güntürkün and Bugnyar, 2016; see discussion below).

Regarding the connections, both the avian/reptilian DVR and the mammalian pallial amygdala receive sensory input from comparable thalamic nuclei (Bruce and Butler, 1984; Butler, 1994a,b; Bruce and Neary, 1995; Guirado et al., 2000). Notably, these nuclei belong to the collothalamus (Butler, 1994b) and locate in the same thalamic compartment: the ventral tier for the auditory-related nuclei (comparable to the mammalian medial geniculate nucleus) and intermediate tier for the visual-related nuclei (including the avian/reptilian nucleus rotundus, which is comparable to the suprageniculate nucleus of mammals; note that the lateral posterior and pulvinar thalamic nuclei – sometimes compared to nucleus rotundus – are located in the dorsal thalamic tier, instead of the intermediate thalamic tier) (Guirado et al., 2000; Puelles, 2001; Puelles and Medina, 2002; Medina et al., 2011, 2017a). In mammals, the collothalamic sensory input targets the lateral and basomedial amygdalar nuclei, while in birds and reptiles, the same thalamic nuclei target segregated areas along the rostrocaudal dimension of the DVR (in birds, these include the entopallium for visual input and field L2 for auditory input; Bruce and Butler, 1984; Bruce and Neary, 1995; Guirado et al., 2000; discussed by Medina et al., 2017a).

In addition to the thalamic input, the pallial amygdala (in particular, the basal nuclear complex of the amygdala, Pessoa, 2008; Medina et al., 2017a) and the avian/reptilian DVR (particularly its posterior part, named posterior dorsal ventricular ridge in lizards, Lanuza et al., 1998; and caudolateral nidopallium in birds, Kröner and Güntürkün, 1999) are extensively connected with other pallial areas, including sensory association centers and are considered high integration centers (discussed by Medina et al., 2017a). Moreover, both project to subpallial areas that include the extended amygdala, which channels descending pathways to similar centers of the hypothalamus and brainstem, involved in the control of endocrine, autonomic, and motor systems (Vicario et al., 2014; Medina et al., 2017a). Notably, both the pallial amygdala (including the basal complex) and the posterior DVR are involved in stress and fear responses and social behavior (reviewed by Medina et al., 2017a). On the one hand, in both birds and mammals, the central extended amygdala areas receiving pallial amygdala-like input (including the arcopallium and parts of the caudal nidopallium and subnidopallium in birds; Atoji et al., 2006; Hanics et al., 2017) project to hypothalamic areas involved in acute and chronic stress responses (Phelps and LeDoux, 2005; Nagarajan et al., 2014; Vicario et al., 2014). Like in mammals, in birds, at least part of the central extended amygdala (lateral bed nucleus of the stria terminalis or BSTL) as well as the pallial amygdala-like areas of the DVR that project to BSTL also become active during stress and are involved in fear behavior (Saint-Dizier et al., 2009; Nagarajan et al., 2014). On the other hand, in birds and mammals, the medial extended amygdala and part of the pallial amygdala become active upon animal exposure to conspecific-related stimuli (olfactory cues in rodents, Choi et al., 2005; visual cues in chicks, Mayer et al., 2017) and are part of the social cognition network (Choi et al., 2005; Mayer et al., 2017).

In spite of these similarities, it is also clear that the pallial amygdala and the DVR also show many dissimilar features regarding cytoarchitecture, chemoarchitecture, connections, and functions (reviewed by Reiner et al., 2004 and Jarvis et al., 2005). These are due to divergent processes during evolution after separation from the last common ancestor. These differences deserve deeper evaluation and more investigation since they can help to understand not only mechanisms behind the evolutionary divergence, but also differences on how different animals process information to solve biologically-relevant problems (similar or different depending on their way of life and their specific ecological and/or social context). For example, rodents are mostly nocturnal animals (with exceptions) and depend highly on olfactory cues for feeding, detecting danger, and social communication, while birds have a relatively less developed olfactory system and rely more on visual and auditory cues (Abellán et al., 2013; Clayton and Emery, 2015). Thus, brain networks and areas dedicated to olfactory or visual processing may show a different degree of elaboration in these different animals. Similarly, brain networks involved in social cognition are expected to show a different degree of elaboration in social versus solitary species and depending on the size of the social group.

## Is There an Orbitofrontal Cortex in Non-Mammals?

In mammals, the orbitofrontal cortex (OFC) is defined as the ventral and oldest part of the prefrontal neocortex (Kringelbach and Rolls, 2004). In human studies, it is often included as part of the medial prefrontal cortex. However, it is located at the frontier between the neocortex and allocortex and shows some peculiarities in cytoarchitecture (with agranular or dysgranular areas, as typical of transition areas) and connections that make it different from the rest of the prefrontal cortex (in primates, this is particularly so for its posterior or limbic part, Barbas, 2007). Regarding the connections, the posterior OFC of primates and comparable areas of rodents (including the infralimbic cortex, among other areas) are reciprocally connected with primary olfactory structures, including the anterior olfactory area and the piriform cortex (Barbas, 1993, 2007; Illig, 2005). The posterior OFC also projects to the main olfactory bulb (Illig, 2005), and there is a report of a very weak direct projection from the main olfactory bulb to a posteromedial part of OFC (the infralimbic cortex) in rats (Neafsey et al., 1986), although the latter has not been confirmed in other studies with rodents (for example, Hintiryan et al., 2012, for the mouse connectome project) or primates (Barbas, 1993; Carmichael et al., 1994). In addition, the posterior OFC is reciprocally connected with the agranular insular cortex (Illig, 2005) and with the basal complex of the pallial amygdala (Ghashghaei et al., 2007; Reppucci and Petrovich, 2016). In contrast, it only shows scarce connections with other parts of the prefrontal cortex (Illig, 2005; Barbas, 2007), such as the anterior and dorsal prefrontal areas showing eulaminar (granular) structure in primates, which display a typical neocortical lamination (Barbas, 2007). The posterior OFC and the basal complex of the pallial amygdala (with which the OFC is reciprocally connected) receive input from visual and auditory association cortices (Barbas, 2007), and both show overlapping projections to the accumbens and adjacent parts of the striatal “emotion processing network” (Heilbronner et al., 2016). The OFC is thus an integration center that is involved in evaluation of the emotional significance of stimuli, in decision-making based on likely-reward, and in mediation of reward- and fear-guided behavior (Elliott et al., 2000). Together with the amygdala, it plays an important role in predicting the reward value of odors (Schoenbaum et al., 1999; Illig, 2005) and other sensory stimuli (Barbas, 2007). In addition, it participates with the amygdala in a network involved in social perception (Bickart et al., 2011, 2012; see also Adolphs, 2003).

The prefrontal cortex – derived from the dorsal pallium – is generally assumed to be an innovation of mammals, although an analogous area (i.e. functionally similar but not homologous) has been described in the caudolateral nidopallium of birds (Güntürkün, 2005). However, the dorsal pallium is quite small in reptiles (Desfilis et al., 2018; Tosches et al., 2018), making unlikely the existence of a homologue of the prefrontal cortex in non-mammals. Interestingly, the OFC was recently proposed to be part not of the neocortex (i.e., dorsal pallium), but of a newly defined pallial sector named DLP (Desfilis et al., 2018). In contrast to the dorsal pallium, the DLP is well developed in birds and reptiles (Abellán et al., 2014; Medina et al., 2017b; Desfilis et al., 2018). This proposal would be partially in line with that posed in the structural model by García-Cabezas et al. (2019), in which they suggest that agranular and dysgranular cortical areas such as those of the posterior orbitofrontal and other limbic cortices (like the agranular and dysgranular insular cortex) are phylogenetically more ancient than eulaminar neocortical areas. In our model, the agranular/dysgranular areas of OFC and insula would be part of DLP, while the eulaminar cortices would derive from the dorsal pallium. In agreement with the structural model, the DLP stands at the base of the dorsal pallium from rostral to caudal levels.

During embryonic development, the mouse DLP is enriched in expression of Nr4a2/Nurr1 and Cadherin 8 (mainly in relation to the claustrum; Medina et al., 2004; Watson and Puelles, 2017) and Cerebellin 2 (Cbln2, preferentially expressed in the insular cortex and OFC) (Allen Developing Mouse Brain Atlas; for OFC see also Figure 1F). The chicken DLP also shows expression of these genes (Figures 1C–E; Reiner et al., 2011; Puelles et al., 2016b), with Nr4a2/Nurr1 and Cbln2 partly segregated to different subdivisions. Notably, in the chicken DLP, expression of Cbln2 extends rostrally into a pallial subdomain intercalated between the hyperpallium and the mesopallium (Figures 1C,E; Reiner et al., 2011). This same subdivision is also poor in Lmo3 (Figure 1A) and was previously called “laminar pallial nucleus” (LAN) due to its relation to the lamina frontalis superior (Suárez et al., 2006). This subdivision is reciprocally connected with the olfactory bulb (Atoji and Wild, 2014), and we suggested that it may represent the avian OFC-homologue (Desfilis et al., 2018). In lizards, the rostral pole of DLP also appears to include an olfactory area (Desfilis et al., 2018) previously identified as part of the anterior olfactory area (Martínez-García et al., 1991). Thus, like in mammals, the OFC-like area of birds and lizards is reciprocally connected with olfactory structures. Moreover, at least in birds, the OFC-like area (the LAN, sometimes identified as the rostral pole of the densocellular hyperpallium) is also reciprocally connected with caudal DVR areas, such as the caudal nidopallium and arcopallium (Kröner and Güntürkün, 1999; Atoji et al., 2006), which are considered homologous to at least part of the pallial amygdala, as explained above (Bruce and Neary, 1995; Puelles, 2001; Martínez-García et al., 2002, 2007; Medina and Abellán, 2009; Medina et al., 2017a; Desfilis et al., 2018; Tosches et al., 2018).

## Brain Networks Involved in Sociality in Mammals: Facts and Model-Based Predictions

**Figure 2:**
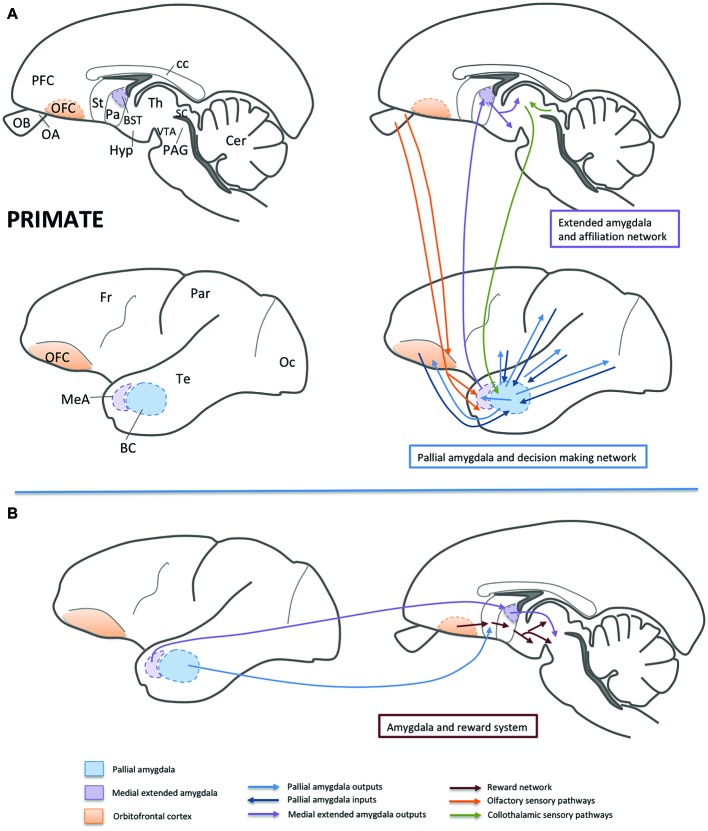
Schemes of parasagittal sections of the primate brain (at different mediolateral levels), representing some of the areas and functional networks related to sociality. For simplicity, only part of the connections is shown. Panel **(A)** shows the networks related to affiliation (involving the medial extended amygdala; i.e., the medial amygdala and BSTM) and to decision-making (involving the basal amygdalar complex of the pallial amygdala and the orbitofrontal cortex). Olfactory and collothalamic sensory inputs are also shown in the schemes. Panel **(B)** shows the influence of the amygdala on the reward system, mainly by way of projections from the pallial amygdala to the ventral striatum and from the BSTM to the ventral tegmental area. Abbreviations: BC, basal amygdalar complex; BST, bed nucleus of the stria terminalis; cc, corpus callosum; Cer, cerebellum; Fr, frontal lobe of NCx; Hyp, hypothalamus; MeA, medial amygdala; NCx, neocortex; OA, anterior olfactory area; OB, olfactory bulb; Oc, occipital lobe of NCx; OFC, orbitofrontal cortex; Pa, pallidum; PAG, periaqueductal gray; Par, parietal lobe of NCx; PFC, prefrontal cortex; SC, superior colliculus; St, striatum; Te, temporal lobe of NCx; Th, thalamus; VTA, ventral tegmental area.

As mentioned above, in humans and other mammals, there are at least two distinct brain networks involved in sociality, whose strength is positively correlated to the size and complexity of the social group: (1) a network related to social perception, involving the pallial amygdala, several neocortical areas (including visual and auditory association areas), as well as the orbitofrontal cortex, and (2) a network related to social affiliation, involving the medial amygdala, some neocortical areas (including parts of the anterior cingulate and prefrontal cortices), the ventromedial striatum (largely including the nucleus accumbens), and the ventromedial hypothalamus (Bickart et al., 2012; see also Adolphs, 2003). Some of these connections are represented in Figure 2. In addition, there is another network involving the central extended amygdala, which is related to fear and aversion, that participates in social aversion (Bickart et al., 2012; see also Adolphs, 2003; and Phelps and LeDoux, 2005).

Regarding the first network that involves ample pallio-pallial connections, the structural model of cortical organization proposed by García-Cabezas et al. (2019) predicts the laminar pattern and strength of the connections of different types of cortices. According to this model, cortical areas with the same lamination type display stronger connections between them than with cortical areas showing a different lamination type. The predictions are confirmed for the case of the OFC, since its agranular/dysgranular parts are reciprocally and strongly connected (with the connections originating and ending in all layers) with similar parts of the insular, perirhinal, and entorhinal cortices (Barbas, 2007; García-Cabezas et al., 2019). This would also agree with predictions of our evodevo-based model, which suggests preferential or stronger connections between areas or cells with the same developmental origin and molecular profile (Medina et al., 2017a). Notably, the agranular/dysgranular areas that are strongly and reciprocally connected locate along the rostrocaudal dimension of the same pallial division, the DLP, according to our model (Desfilis et al., 2018).

The structural model also predicts that connections between cortices of different lamination types would be weaker and involve only part of the layers, and this would apply for the connections between OFC with the visual and auditory association neocortical areas: the projections originate in deep layers of agranular/dysgranular OFC and terminate in superficial layers (specially layer I) of eulaminar cortices, and the projections from eulaminar cortices to agranular/dysgranular OFC originate in superficial layers and terminate in middle layers (García-Cabezas et al., 2019). The structural model does not extend predictions to the connections between cortical areas and the pallial amygdala, since the latter does not show a laminar organization. New experimental data in rats have shown that in the basolateral amygdala, different neurons project to either the prefrontal cortex (including the infralimbic cortex) or the lateral hypothalamus (Reppucci and Petrovich, 2016). Developmental studies in mouse showed that the basolateral amygdala includes neurons of at least two different lineages, Emx1 and non-Emx1 (the latter are the typical ventral pallial cells that belong to Dbx1 and/or Lhx9 lineages, as reviewed by Medina et al., 2017a). Our evodevo-based model of six pallial divisions would predict that cells of the basolateral pallial amygdala that project to the prefrontal cortex would preferentially belong to Emx1 lineage (and would target a region rich in Emx1-lineage cells), a proposal that requires investigation. Thus, our evodevo-based model of six pallial divisions represents a more ample framework for understanding the pallium and for predicting connections between its subdivisions, which can extrapolate to the pallium of non-mammals to evaluate the degree of conservation or variation, offering new venues for a better comprehension of the organization and evolution of functional networks involving the pallium.

## Brain Networks Involved in Sociality in Non-Mammals

**Figure 3:**
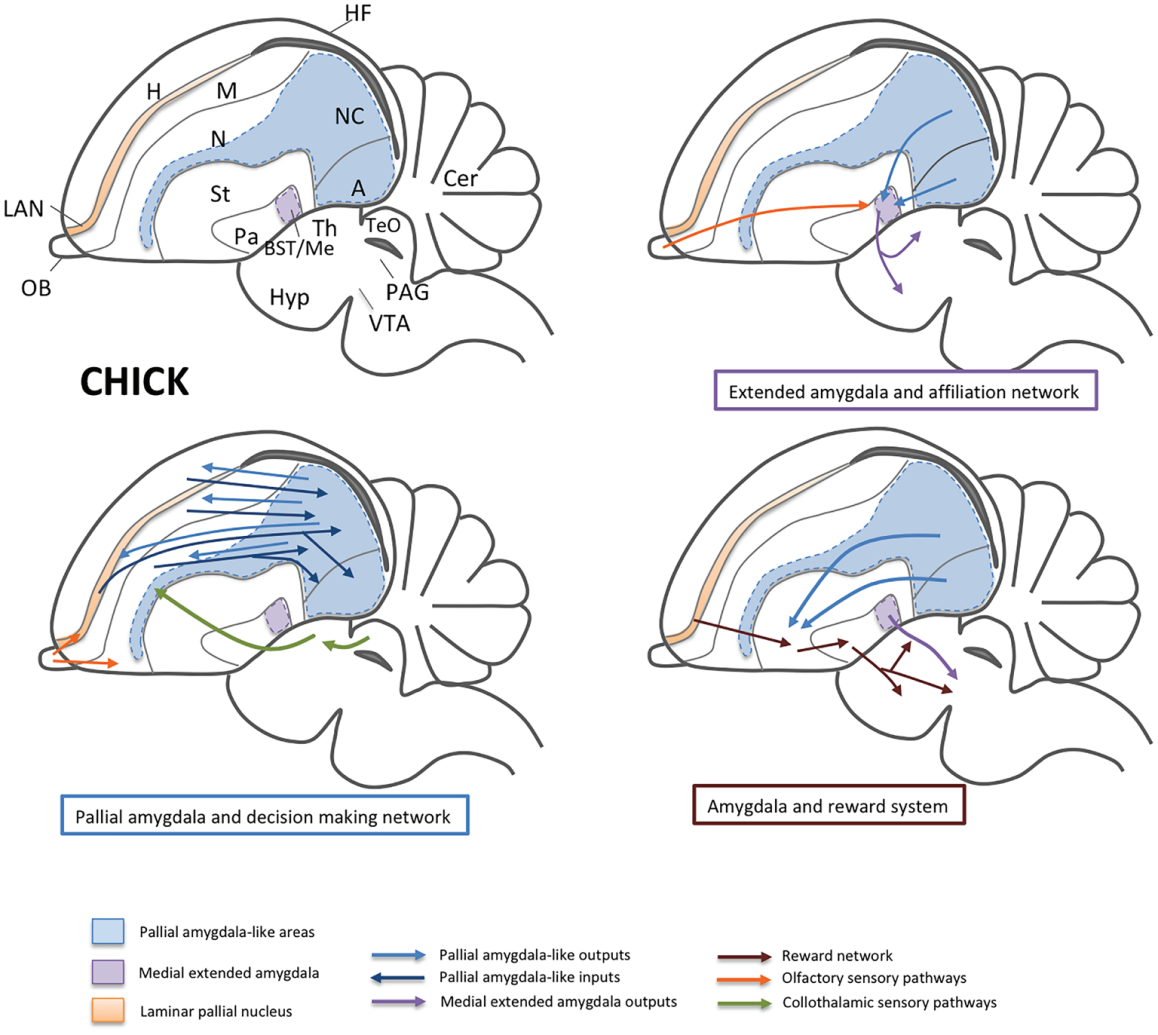
Schemes of a lateral view of the chick brain, representing some of the areas and functional networks related to sociality. For simplicity, only part of the connections is shown. Separate schemes show the networks related to affiliation (involving the medial extended amygdala; i.e., the medial amygdala and BSTM) and to decision-making (involving the nidopallial and arcopallial areas comparable to the pallial amygdala, and the LAN, comparable to the orbitofrontal cortex). Olfactory and collothalamic sensory inputs are also shown in the schemes. In a separate scheme, the influence of the amygdala on the reward system is represented, mainly by way of projections from the pallial amygdala to the ventral striatum and from the BSTM to the ventral tegmental area. Abbreviations: A, arcopallium (ventrocaudal pallium); APH, parahippocampal area; BST, bed nucleus of the stria terminalis; Cer, cerebellum; DLP, dorsolateral pallium; H, hyperpallium (dorsal pallium); Hyp, hypothalamus; LAN, laminar pallial nucleus (rostral part of DLP comparable to the orbitofrontal cortex); M, mesopallium (lateral pallium); Me, medial amygdala; N, nidopallium (ventral pallium); NC, caudal nidopallium; OB, olfactory bulb; Pa, pallidum; PAG, periaqueductal gray; St, striatum; TeO, optic tectum of the midbrain (comparable to the superior colliculus); Th, thalamus; VTA, ventral tegmental area.

Are there networks in non-mammals similar to those of mammals involved in sociality? Although more neuroethological studies are needed, the answer is yes (Figure 3). However, there are differences in these networks between mammals and non-mammals. Regarding the network involving the pallial amygdala, while in mammals, the predominant reciprocal connections are with the neocortex (dorsal pallium) and OFC (noted above to be part of DLP), in sauropsids, the pallial areas primarily interconnected with the amygdala are not only neocortical-like (dorsal pallium; Wulst or hyperpallium in birds) and OFC-like (the LAN in rostral DLP), but also include parts of other pallial sectors such as the LP (mesopallium). However, in birds and, especially, in reptiles, most of the sensory association information (visual, somatosensory, and auditory) is processed and transmitted within the ventral pallium, from rostral/intermediate to caudal parts (Lanuza et al., 1998; Kröner and Güntürkün, 1999; Güntürkün, 2005). In particular, the anterior DVR of birds and reptiles include different nuclei that receive sensory input directly from the collothalamus or the brainstem (Butler, 1994a,b). The DVR thalamorecipient nuclei have been compared to the thalamorecipient lateral nucleus and/or the basomedial nucleus of the mammalian basal amygdalar complex (Bruce and Neary, 1995; Medina et al., 2017a; and Desfilis et al., 2018), a conclusion also partially supported by data from single cell transcriptome (which shows high similarity between glutamatergic cells of anterior DVR and lateral amygdalar nucleus; Tosches et al., 2018). These sensory nuclei of the DVR project to adjacent association areas, which in turn project to the caudal or posterior DVR (Lanuza et al., 1998; Kröner and Güntürkün, 1999). This is where the most important high integration centers are located in sauropsids: the caudal nidopallium and arcopallium in birds and the equivalent parts in the posterior DVR of reptiles (Lanuza et al., 1998; Kröner and Güntürkün, 1999; Güntürkün, 2005). Based on similarity of connections and important role in cognition (including decision-making and working memory), the caudolateral nidopallium of birds have often been compared to the prefrontal cortex of mammals (Kröner and Güntürkün, 1999; Güntürkün, 2005; Güntürkün and Bugnyar, 2016), but if we consider the topological location, embryonic origin and gene expression profile, together with some of the connections, it becomes clear that the avian caudal nidopallium (and posterior DVR of lizards) is part of the ventral pallium and at least partially homologous to the basal complex of the amygdala of mammals (Lanuza et al., 1998; Puelles, 2001; Medina et al., 2017a). However, the pallial amygdala is also part of the same functional network that, together with the prefrontal cortex, plays a critical role in cognition, including decision-making. Therefore, another way of viewing the avian caudolateral nidopallium (and the corresponding high integration area of the lizard posterior DVR; Lanuza et al., 1998) is as a distinct pallial amygdala-like caudal area that work together with a prefrontal-like rostral area as part of a functional network involved in cognition (as discussed by Medina et al., 2017a).

Nevertheless, the homology of DVR with basal complex of the amygdala is only partial, since the degree of enlargement and elaboration of the DVR is very high in extant reptiles, and especially in birds, and includes multiple subdivisions and complex connections between them. In addition, the basal complex of the amygdala is also very large in some mammals, such as primates, but this enlargement has been accompanied by a great expansion and elaboration of areas of the neocortex (dorsal pallium) with which the mammalian pallial amygdala is reciprocally connected. As a consequence, some of the connections that reach the basal complex of the amygdala in mammals and the caudal/posterior DVR in sauropsids likely evolved independently. This is particularly so for part of the auditory input, which directly reaches a specific area of the DVR in sauropsids (field L2 in the caudomedial nidopallium of birds) and the lateral/basomedial nuclei of the basal amygdalar complex of mammals, originating from a homologous medial geniculate-like nucleus of the collothalamus (reviewed by Medina et al., 2017a). In addition, in mammals, high order auditory information also reaches the basal complex of the amygdala from an association area of the temporal neocortex, but this second link is apparently missing in sauropsids. In birds, the auditory information is processed in secondary association areas of the nidopallium adjacent to field L2, and from here it is transmitted to the caudolateral nidopallium and arcopallium (in the posterior DVR), where it is integrated with information of other modalities coming from different pallial divisions (Kröner and Güntürkün, 1999).

As noted above, both the caudal nidopallium and the arcopallium (including the so-called posterior pallial amygdala according to Reiner et al., 2004; and Atoji et al., 2006) are reciprocally connected with a thin area intercalated between the hyperpallium and the mesopallium (Kröner and Güntürkün, 1999; this is included as part of the densocellular hyperpallium by Atoji et al., 2006), an area proposed by us to be comparable to the mammalian OFC (Desfilis et al., 2018). The mammalian OFC plays an important role in decision-making, including choices in social contexts (Campbell-Meiklejohn et al., 2012; Watson and Platt, 2012; Xia et al., 2015). In mammals, it appears that the brain makes simple choices by assigning value to the options under consideration, and the pallial amygdala-to-OFC projection is essential for this and more important than the reciprocal OFC-to-amygdala projection (Jenison, 2014). In birds, neurons of the caudolateral nidopallium display value-related activity (Dykes et al., 2018). In addition, the caudal nidopallium plays a role in decision-making, and at least in crows is involved in complex tasks using flexible working memory (Güntürkün, 2005; Güntürkün and Bugnyar, 2016; Hartmann et al., 2018). Regarding social contexts, mate choice decisions are extremely important because they affect the genetic constitution of offspring and, in some species, the quality/quantity of parental care (Sockman, 2007). Songbird females make mate choices based on song features (Sockman, 2007). In European starlings, females prefer long songs, and these induce higher ZENK activation in the caudomedial mesopallium and in the auditory-related caudomedial nidopallium (Sockman, 2007). Moreover, enriched social contexts induce an increment in neurogenesis in the caudal nidopallium (Lipkind et al., 2002). However, it is unclear whether the projection from the caudal nidopallium to the OFC-like area plays a role in making choices in social contexts. Regarding other areas of the network, the arcopallium does not appear to be involved in food selection-related choices (Aoki et al., 2006b), but the lateral arcopallium has been shown to contribute to social facilitation of foraging efforts in domestic chicks (Xin et al., 2017). More studies are needed to evaluate the implication of the arcopallium in social choices.

The caudal nidopallium, subnidopallium, and arcopallium (and the equivalent areas in reptiles) project to the extended amygdala and striatum (Lanuza et al., 1998; Kröner and Güntürkün, 1999; Atoji et al., 2006; Hanics et al., 2017). In particular, the arcopallial descending projections target the medial striatum (comparable to the nucleus accumbens and related to reward) (Aoki et al., 2006a; Hanics et al., 2017). Like in mammals, the projection through the medial striatum may modulate reward, including social reward (O’Connell and Hofmann, 2011; Frith and Frith, 2012; Dölen et al., 2013). In songbirds, nidopallium and arcopallium are part of sophisticated brain networks involved in song learning and production, which are fundamental for social interactions in these species (reviewed by Jarvis et al., 2013; Pfenning et al., 2014). Male vocalizations, both sexually motivated (to attract females) and “undirected,” require the involvement of the reward system, although different patterns of dopamine activity and opioid release are involved (Riters, 2012; Riters et al., 2017).

The pallial amygdala descending projection through the extended amygdala relates to other aspects of social behaviors and to emotional responses (reviewed by Martínez-García et al., 2007; Medina et al., 2017a). In the extended amygdala of birds and reptiles, it is possible to distinguish between the central extended amygdala and the medial extended amygdala (Abellán and Medina, 2009; Vicario et al., 2014, 2015, 2017; Medina et al., 2017a). Like in mammals, the central extended amygdala (including the lateral bed nucleus of the stria terminalis or BSTL) has been associated to fear responses, stress, and aversion (Saint-Dizier et al., 2009; Nagarajan et al., 2014), while the medial extended amygdala has been related to different aspects of social behavior, including affiliation, sexual, and agonistic behaviors (reviewed by Martínez-García et al., 2007; Medina et al., 2017a). Notably, in chicken, the arcopallium includes areas projecting to the medial extended amygdala, which become active following visual exposition of naive animals to conspecifics (Mayer et al., 2017). In agreement with this observation, the arcopallium contains many cells that respond to certain color and shape stimuli (Scarf et al., 2016). The visual information seems to reach the arcopallium indirectly by way of the collothalamic pathway; the information first reaches the entopallium and is then transmitted to the arcopallium by intratelencephalic projections (as discussed by Mayer et al., 2017). The arcopallium also projects to the tectum, closing a tecto-tectal loop, which like in humans may be involved in early social orienting responses (Mayer et al., 2017).

Like in mammals, in birds and reptiles the medial extended amygdala (including the medial bed nucleus of the stria terminalis or BSTM) controls social behavior by way of projections to the preoptic area and hypothalamus (reviewed by Martínez-García et al., 2007; Medina et al., 2017a). In mammals, this pathway is part of the network involved in affiliation (Bickart et al., 2012), which is modulated by vasopressin, oxytocin, and their receptors (Hammock and Young, 2006; Donaldson and Young, 2008; Goodson, 2013). The affiliative behavior is mediated by the central projections of vasopressin and oxytocin containing cells, located mainly in the supraoptic (SO) and paraventricular (PVN) hypothalamic nuclei, as well as in the medial extended amygdala (primarily the BSTM) (De Vries and Miller, 1998). These cells project to the amygdala (including the basolateral complex, central and medial nuclei), but have also projections to brain areas of the reward network (including the nucleus accumbens, the ventral tegmental area, and the prefrontal cortex), the hippocampal formation (related to memory formation), the septum, and the anterior olfactory area (Walum and Young, 2018). Vasopressin (vasotocin, VT) and/or oxytocin (OT) receptors are found in all these areas, but there are significant variations of their expression in species exhibiting differences in pair-bonding, such as the monogamous prairie voles versus the non-monogamous meadow and montane voles (Hammock and Young, 2006; Donaldson and Young, 2008). The nucleus accumbens, the ventral pallidum, the prefrontal cortex, and the amygdala are among the areas showing higher receptor density in monogamous compared to non-monogamous voles (Insel et al., 1994; Young and Wang, 2004). Intra-cerebroventricular infusion of OT facilitates partner preference formation in both sexes, and this seems to be mediated through the reward system (Walum and Young, 2018). It appears that social interactions facilitate dopamine release from axon terminals in the nucleus accumbens and prefrontal cortex (the dopaminergic input comes from the ventral tegmental area), as well as OT release in multiple areas including those of the reward system (reviewed by Walum and Young, 2018). Blocking OT receptors in the nucleus accumbens or the prefrontal cortex (both part of the reward network) prevents the formation of mating-induced partner preference (Walum and Young, 2018). However, striking individual variations are also found within monogamous prairie voles regarding the density of oxytocin receptors in nucleus accumbens (Walum and Young, 2018). It appears that a high density of oxytocin receptors in this nucleus confers resilience to neglect in a paradigm involving neonatal social isolation and relates to the ability to form partner preferences later in life (Walum and Young, 2018). Regarding vasopressin, it plays a role in social recognition, territorial scent marking, and aggressive behaviors (Walum and Young, 2018). In prairie vole males, it facilitates pair-bonding, which is mediated by VT1a receptors. These show higher density in the ventral pallidum of the monogamous species compared to the non-monogamous ones, and blocking VT1a receptors in the ventral pallidum of the monogamous prairie voles inhibits formation of partner preference (Walum and Young, 2018). VT1a signaling in the lateral septum is also important for some aspects of pair-bonding and is possibly related to social recognition. However, VT1a receptors show higher expression in the septum of non-monogamous species of voles than in the monogamous one (Walum and Young, 2018).

While only 9% of mammals are socially monogamous, most birds (90%) are monogamous (although this does not imply sexual exclusivity, for example Dolan et al., 2007; Lukas and Clutton-Brock, 2013). In birds, the SO, PVN, and BSTM also include populations of vasopressin (vasotocin) and oxytocin (mesotocin) cells (Aste et al., 1998; Panzica et al., 1999; De Vries and Panzica, 2006; Vicario et al., 2017). Like in rodents, in songbirds, there are striking interspecific and intraspecific variations in the expression of vasopressin and oxytocin receptors (Leung et al., 2011). In zebra finch, different VT-like and OT-like receptors are found in parts of the nidopallium and arcopallium, including auditory and vocal control related areas (Leung et al., 2011). In addition, in zebra finch and white-throated sparrow, VT-like receptors (including VT1a-like or VT4) are found in the septum, parts of the reward system (medial striatum, ventral pallidum, ventral tegmental area), and part of the medial extended amygdala (BSTM) (Leung et al., 2011). Moreover, OT-like receptors (VT3) are found in the hippocampus in sparrows, although not in that of finches (Leung et al., 2011). Activation of oxytocin and vasopressin 1a like receptors increases partner preference and/or gregariousness in zebra finches (Goodson, 2013). Vasopressin also increases aggressive competition for mates in finches (Goodson, 2013). It is likely that the action of these peptides is mediated in part through the reward system, although it may involve different receptors (through cross-binding of oxytocin and vasopressin to several of them). In songbirds, many pallial areas of the anterior and posterior circuits controlling song learning and production express VT and OT receptors, and it is likely that these peptides modulate social interactions through these neural systems as well.

In conclusion, birds and reptiles share with mammals some networks of those described in mammals for social perception, salience, and decision-making (the network including the pallial amygdala) and for social affiliation and recognition (the network including the medial amygdala). At least in birds, these networks play roles resembling some of those described in mammals, such as decision-making (including social contexts) and affiliation. However, while some of the connections of the networks likely derived from those present in the common ancestor, others appear to have evolved independently. More studies are needed to better understand the role of these networks in social cognition and behavior in birds (and reptiles), as well as the similarities and differences of distinct subcomponents with those found in different mammals. One interesting similarity between birds and primates relates to the fact that the networks in birds preferentially involve visual and auditory cues, and in songbirds include different areas of the auditory and song control systems. Finally, it is important to remark that in mammals, birds, and reptiles, there is an important interaction between the different networks by way of cross projections between areas of both systems (for example, see O’Connell and Hofmann, 2011). Future functional studies need to address the consequences of the anatomical cross talks observed between these networks.

## Data Availability

All datasets generated for this study are included in the manuscript and/or the supplementary files.

## Author Contributions

All authors contributed significantly to the research and ideas that led to preparation of this article. LM prepared the first version of the manuscript. ED and AA discussed and revised it. AA prepared the *in situ* hybridization material shown in Figure 1. LM and ED prepared the schemes shown in Figures 2, 3.

### Conflict of Interest Statement

The authors declare that the research was conducted in the absence of any commercial or financial relationships that could be construed as a potential conflict of interest.
